# Effects of a Short Emotional Management Program on Inpatients with Schizophrenia: A Pilot Study

**DOI:** 10.3390/ijerph18105497

**Published:** 2021-05-20

**Authors:** Kyung-Hwan Park, Eun-Sook Park, Sung-Mi Jo, Mi-Hui Seo, Young-Ok Song, Sun-Joo Jang

**Affiliations:** 1 National Center for Mental Health, 127, Yongmasan-ro, Gwangjin-gu, Seoul 04933, Korea; siloam02@korea.kr (K.-H.P.); pepe914@korea.kr (E.-S.P.); smcho0907@hanmail.net (S.-M.J.); satsang@korea.kr (M.-H.S.); syonbmh@korea.kr (Y.-O.S.); 2 Red Cross College of Nursing, Chung-Ang University, Seoul 06974, Korea

**Keywords:** schizophrenia, emotion, inpatient, negative symptom, nursing

## Abstract

The prevalence of schizophrenia is gradually increasing worldwide. Many patients with schizophrenia have a diminished ability to empathize and to detect their own emotions or those of others, deteriorating their social functioning and their quality of life. Nonetheless, emotional management training may improve patients’ emotion recognition, emotional expression, and negative symptoms. Developing and applying a short but effective program that reflects the current medical environment, in which hospital stays are ever-diminishing, is warranted. This one-group, pretest–posttest, quasi-experimental pilot study aimed to examine the effects of a short emotional management program (EMP-S) on 17 patients with chronic schizophrenia. Participants were patients hospitalized in the National Center for Mental Health in Korea. After the completion of a twice-a-week, eight-session, four-week long EMP-S, participants showed improvements in emotion recognition, emotional expression, and negative symptoms. Our results suggest the applicability and potential effectiveness of the EMP-S, which takes the length of psychiatric hospital stay and the inpatient environment into consideration. To minimize any barriers to social functioning in the post-discharge lives of inpatients with chronic schizophrenia and enhance their social cognition—by improving their emotion recognition, emotional expression, and negative symptoms—we suggest the periodical administration of this EMP-S to these inpatients.

## 1. Introduction

Schizophrenia is a chronic and serious mental disorder affecting more than 19,776,900 people worldwide [1]. Individuals with schizophrenia often experience challenges at work and/or in their family relationships owing to positive symptoms (e.g., delusions, hallucinations, and disturbed language and behaviors), as well as negative symptoms such as affective flattening, which in turn deteriorate their quality of life [2]. Moreover, the years lived with disability (YLD) for schizophrenia is slowly increasing [1]. YLD is a construct that refers to the burden of disease and can be calculated as the product of a prevalence estimate and a disability weight for health statuses [1]. A meta-analysis on empathy and interpersonal relationships in schizophrenia observed that individuals with this disorder have poorer empathic concern and perspective-taking, and higher interpersonal distress compared with healthy controls [3]. Additionally, a majority of patients with schizophrenia have difficulties in detecting minute differences in neutral, positive, and negative stimuli, and in recognizing their emotions and responding accordingly [4]. They also have an impaired ability to read or empathize with others’ emotions, and utilize inappropriate emotional expression and communication methods [3,5]. These symptoms are one of the factors that undermine the social functioning of such individuals [6].

In middle- and low-income countries, approximately 69% of patients with schizophrenia are not treated properly [7]; furthermore, even if the acute clinical symptoms of schizophrenia can be effectively alleviated with cost-effective pharmacological therapies, these treatments have limitations in improving individuals’ social, cognitive, and emotional functions [8]. On the topic, a study remarked that the goal of psychiatric rehabilitation programs should be to help patients develop appropriate social responses that allow for them to carry on their lives as independent members of society; these programs could provide such opportunities by addressing the emotional and social problems mentioned above, ultimately helping them improve their interpersonal relationships [9].

According to some studies on the negative symptoms of schizophrenia [10,11], factor analyses of the most used evaluation scales for these constructs showed that negative symptoms are classified into two categories: emotional expression and motivation/pleasure. Beck et al. [12] reported that, early after onset, patients with schizophrenia have diminished social cognitive functions—including emotion recognition (further defined below)—which repeatedly exposes them to disappointments and frustrations in social settings; as a result, they develop a negative attitude toward social life, followed by a loss of desire and interest, thereby leading to the typical negative symptoms that characterize this disorder. These symptoms, in turn, affect patients’ functional recovery, denoting that improving them is crucial when attempting to effectively treat patients with schizophrenia [13].

Emotion recognition refers to the ability to detect one’s or others’ inner emotions and to recognize and evaluate complex and subtle emotions [14]; emotional expression, on the other hand, refers to the ability to express one’s emotions using words or behaviors appropriate to the context [15]. According to a meta-analysis on emotions in patients with schizophrenia [16], these patients more frequently experience negative (e.g., depression), rather than positive, emotions compared to healthy controls. Moreover, a study using emotion attribution tasks revealed that these patients have a deficiency in their “theory of mind,” denoting that they have a poor ability to empathize with others [17]. Furthermore, patients with schizophrenia also have poor emotion recognition and awareness, which deteriorates their ability to comprehensively grasp a social situation and function accordingly [18]. Although recognizing others’, and expressing one’s, emotions is crucial for the successful social adjustment of patients with schizophrenia [19], studies on the emotional management of patients with schizophrenia have been largely limited in their scope.

Herein, we present some of the evidence that can be found on the topic in the scientific literature. A study showed that emotion recognition and expression in patients with schizophrenia can be improved with professional training [20]. Further, an emotional management program (EMP) administered in a clinical setting was shown to improve the emotional expression, personal relationships, and social behaviors of patients with schizophrenia [21,22]; another similar study showed that EMP enhanced their empathy, brought about positive changes in their personal relationships, and improved their quality of life [22]. Moreover, a study employing a shortened version of an EMP for patients with chronic schizophrenia, in a facility that helps with adjustment to community, confirmed that it improved patients’ emotion recognition, emotional expression, and quality of life [23].

Psychiatric institutions, under the influence of stringent utilization management, are beleaguered to reduce psychiatric inpatient length of stay to show that they are rapidly and cost-effectively treating patients [24,25]. However, when discussing the treatment of schizophrenia patients, focus should not be placed only on reducing the length of stay owing to economic reasons; above all, the development and application of a short, but effective, treatment module for the treatment and rehabilitation of psychiatric patients should be guaranteed [26].

Thus, considering the clinical feasibility of the program related to the length of stay of patients with schizophrenia in mental hospitals, we further reduced the duration of the shortened version of an EMP proposed in a prior study [23]; we modified the original once-a-week, eight-week EMP to a twice-a-week, eight-session, four-week EMP (hereinafter named EMP-S). Specifically, this pilot study served to examine the effectiveness of the EMP-S and thereafter finalize it; after the termination of its development, it is supposed to serve as a nursing intervention to promote the recovery of patients with schizophrenia.

### Aim

This study aimed to investigate the applicability of the EMP-S, expecting improvements in emotion recognition, emotional expression, and negative symptoms of inpatients with schizophrenia.

## 2. Materials and Methods

### 2.1. Design and Participants

This pilot study employed a one-group, pretest–posttest, quasi-experimental design. The sample size was determined using G-power 3.1.9; with reference to a previous study [21], the conditions for repeated measures ANOVA were set to an effect size (f) of 0.40, significance of 0.05, and power of 0.90. The minimum sample size was computed to be 15; considering a 20% withdrawal rate, we recruited 19 participants. Study participants were inpatients with schizophrenia at the National Mental Hospital of Korea.

#### 2.1.1. Inclusion Criteria

- adults aged 19–64 years;
- diagnosis of schizophrenia based on the DSM-5 and ICD-11 by a psychiatrist;
- absence of organic brain syndrome assessed through brain imaging, electroencephalography, and laboratory tests;
- ability to read and respond to the questionnaire and understand the contents of the program;
- a score of 40 or higher in the Global Assessment of Functioning (GAF) Scale—this scale was chosen by referencing the study by Fenger et al. [27], and served to assess patients’ global psychological, social, and occupational functioning;
- deemed to be fit to participate in the program by their doctor;
- signing of the written informed consent.

#### 2.1.2. Exclusion Criteria

- adults aged over 65 years;
- a score of under 40 on the GAF Scale;
- difficulty in reading and responding to the questionnaire and understanding the contents of the program;
- organic mental disorders or intellectual disability.

### 2.2. Measures 

#### 2.2.1. Emotion Recognition

Emotion recognition was measured using the self-reported, 22-item, short Korean version of the Trait Meta-Mood Scale. It comprises emotional attention and emotional clarity subscales. Responses are marked on a 5-point Likert-type scale, with total scores ranging from 22–110 and higher scores indicating a clearer recognition of, and focus on, one’s emotions. This scale has been validated for use among Korean patients with schizophrenia by Cha [28], and it was developed based on an original 48-item scale used to measure emotional intelligence in psychiatric patients (Cronbach’s α ranging from 0.82–0.87) [29]. In this study, Cronbach’s α was 0.79.

#### 2.2.2. Emotional Expression

Emotional expression was measured using the self-reported, 16-item Berkeley Expressivity Questionnaire [30]. It comprises the positive expressivity, negative expressivity, and impulse strength subscales. Responses are marked on a 5-point Likert-type scale, with total scores ranging from 16–80 and higher scores indicating a higher emotional expression. At the time of development, the Cronbach’s α of the tool was 0.81 [30]; in this study, it was 0.74.

This tool has been widely used for measuring emotional expression in patients with schizophrenia [31], and it specifically measures individual differences in the outer expressions of individuals’ emotional experiences.

#### 2.2.3. Negative Symptoms

Negative symptoms were measured using the 25-item Scale for the Assessment of Negative Symptoms (SANS) [32], which is one of the most well-known and widely used tools in clinical practice to assess negative symptoms [33]. It comprises the following five subscales: affective blunting, alogia, apathy, asociality, and attention. It is responded to by a mental health professional, with total scores ranging from 0–150 and higher scores indicating greater negative symptoms. At the time of development, the Cronbach’s α of the tool was 0.86; in this study, it was 0.96.

### 2.3. EMP-S

The intervention comprised eight sessions, with two sessions for each of the four aspects of emotion that were addressed (emotion recognition, emotional expression, emotional utilization, and emotional regulation), and was administered twice-a-week for four weeks; this modified procedure served to shorten the existing EMP, which administered one session per week for eight weeks [23]. We also modified the contents of the EMP-S, tailoring them to inpatients; specifically, the key contents of each aspect of emotion that needed to be dealt with are described in Table 1. The validity of the EMP-S was examined by six experts (i.e., two clinical psychologists, two psychiatric nursing professors, and two psychiatric nurses), yielding a content validity index (CVI) of 0.91.

### 2.4. Data Collection

Of the 19 participants that were recruited, two withdrew from the study (10% withdrawal rate), resulting in 17 participants that were included in the EMP-S and whose data were analyzed. Considering the average days of hospital stay and the optimal number of participants required to be in the EMP-S—which was a group treatment intervention—we conducted a four-week program for different participants at different time points (i.e., owing to different participants having different days of hospital stay), forming two groups; each group comprised eight–nine participants. The EMP-S was conducted in these groups between November 2018 and January 2019. General characteristics, disease-related characteristics, and emotional cognition and emotional expression scores were measured using a self-report questionnaire that was administered immediately before beginning the program (i.e., pre-test), two weeks into the program, and immediately after the completion of the program (post-test). The scores for the GAF and SANS were measured by a psychiatric nurse practitioner who was not involved in the conduction of this study.

### 2.5. Data Analysis

The collected data were statistically processed and analyzed using SPSS, version 24.0. After analyzing the general characteristics through descriptive statistics, we analyzed participants’ score changes during (i.e., two weeks into the program) the program and post-test by comparing them with pre-test scores using generalized estimating equations (GEEs) with an autoregressive correlation structure. The data were repeatedly measured, were clustered, and were correlated; however, the GEEs do not assume the independence and homogeneity of variance, so we can adjust for individual and interaction confounders [34]. To test tool reliability, we computed their Cronbach’s α coefficients.

### 2.6. Ethical Considerations

This study was approved by the institutional review board of the National Center for Mental Health (116271-2018-50)—to which all authors are affiliated, except for SJJ—and the investigations were carried out following the rules of the Declaration of Helsinki of 1975, revised in 2013. All participants received the following information: study rationale, purpose, and procedure. They were guaranteed that the collected data would be processed anonymously, the data would be used solely for research purposes, and that they had the freedom to withdraw from the study at any time. Further, a written informed consent form was provided to all participants, and all were requested to sign it prior to participation.

## 3. Results

### 3.1. General Characteristics and Disease-Related Characteristics

In total, 64.7% of the participants were women, and the most common age group was 50–59 years (58.5%). Most (53.9%) participants had an associate degree or higher, and the most common duration of disease was 10 years or longer (47.0%). In total, 70.6% of the participants had a history of four or fewer admissions to a psychiatric hospital. Regarding employment, 64.7% had never had a job and 17.6% had maintained a job for two years or longer. All of the participants’ duration of untreated psychosis was less than one month and timely medicated (Table 2).

### 3.2. Comparison of Dependent Variables According to General and Disease-Related Characteristics

There were no significant differences in emotion recognition, emotional expression, and negative symptoms by sex, age, education, duration of disease, number of psychiatric hospital admissions, and duration of employment. However, emotional expression and negative symptoms differed according to GAF scores; specifically, participants who showed scores above 51 for the GAF had higher emotional expression scores (*t* = 2.41, *p* = 0.031) and lower negative symptom scores (*t* = 3.85, *p* = 0.002) than those with a score of less than 50 for the GAF (Table 3).

### 3.3. Verification of the Effects of the EMP-S

#### 3.3.1. Emotion Recognition

The emotion recognition score significantly increased two weeks into the program and in the post-test as compared to the pre-test. The scores were B (Standard Error [SE]), 3.07(2.09) at two weeks and 7.00(2.60) post-test, showing that the estimated mean of the emotion recognition score increased by 3.07 at two weeks and by 7.00 at post-test compared with the pre-test. The score changes were statistically significant (Wald’s test = 14.03, *p* = 0.001; Table 4).

#### 3.3.2. Emotional Expression

Although the emotion expression score increased two weeks into the program and in the post-test as compared with the pre-test, the increase was not statistically significant (Wald’s test = 4.15, *p* = 0.126). The estimated mean increased by 1.48 at two weeks into the program (B[SE] = 1.79[1.48]), which was a statistically significant change (Wald’s test = 1.46, *p* = 0.046). When the pre-test score was compared to the post-test score, however, there was no statistically significant change (B[SE] = 3.66[1.83]) (Table 4).

#### 3.3.3. Negative Symptoms

Negative symptom scores significantly declined at two weeks into the program and at the post-test compared to the pre-test. The negative symptom score was B(SE), −24.20(3.11) at two weeks into the program and −44.87(4.31) at post-test, showing that the estimated mean decreased by 24.20 at two weeks into the program and by 44.87 at post-test compared with pre-test. The score changes were statistically significant (Wald’s test = 115.27, *p* < 0.001; Table 4).

## 4. Discussion

In the current study, after conducting EMP-S for inpatients with schizophrenia twice a week, with a total of eight sessions for four weeks, the emotion recognition score significantly increased, and the negative symptom score significantly decreased. While the emotional expression scores increased, they were not statistically significant.

One difference regarding the results of this study compared to previous research is that the EMP was shortened, lasting only four weeks; this adds to the significance of this study because we were able to verify whether the EMP-S is applicable to the current environment of psychiatric hospitals, in which the duration of inpatient care has shortened. We also attempted to examine the timing at which the effects of the EMP would start to take place.

Patients with schizophrenia who participated in the EMP-S showed a gradual increase in their emotion recognition score at two (i.e., a test conducted midway into the program) and at four weeks into the program (i.e., post-test) compared with baseline (i.e., pre-test), and the changes were statistically significant. This is consistent with the findings of Cho and Jang [23], which showed that the EMP improved sensitivity to facial expressions, emotional attention, and emotional clarity in patients with chronic schizophrenia. Another study also corroborates our results, which showed that the EMP improved the emotion recognition skills regarding negative emotions in patients with this condition [35]. Moreover, our evidence shows that the emotion recognition score began to significantly improve at two weeks into the program, showing that the potential effects of the program manifest relatively quickly. These results suggest that the EMP-S that we proposed has the potential to be applied and may be useful in hospital settings for patients with schizophrenia who have poor emotion recognition and a shortened duration of inpatient care [5].

Further, the emotional expression score tended to improve at two and at four weeks into the program, although these changes were, generally, not statistically significant. This contradicts the literature; studies by Won [21], and Cho and Jang [23]—which both conducted a once-a-week, eight-week program—and Park and Park [36]—which conducted a twice-a-week, eight-week program—observed improvements in the emotional expression of patients with chronic schizophrenia. Upon analysis, we noted that there is a clear difference between the duration of the intervention we proposed and that of these previous studies, suggesting that it would take more than four weeks to foster the emotional expression of this specific group of patients. Furthermore, a study on the emotions of patients with schizophrenia [37] proposed that patients can be divided into the following three groups based on their clinical representations: one with a lack of joy, another with difficulty in emotional regulation, and another with difficulties in emotional expression; namely, some participants in this cited study were likely to have difficulties in emotional expression, such as in our study. This denotes that future studies are warranted to examine patients’ clinical representations prior to the design of the program; this may allow for the development of interventions that are tailored to the specific characteristics of the target group of patients with schizophrenia.

Additionally, negative symptoms gradually improved at two and at four weeks into the program compared with baseline, and the changes were statistically significant. Although we only used one group in this study—hindering our ability to directly confirm the potential effects of the EMP-S owing to the lack of a control group—negative symptoms in our sample seem to have been influenced by interactions with others whom patients encountered while participating in the program/intervention provided by the therapist. On the topic, a study showed that to improve negative symptoms, it is important to have patients express their emotions and increase their interactions with others through activities, such as role-play [38]. Accordingly, the focus of our proposed EMP-S—on interpersonal interactions and emotional expression—seems to have contributed to its effectiveness. Further, considering that all five negative symptoms significantly improved at two and at four weeks into the program, the potential effects of the EMP-S seem to have started early on into the program; despite these positive results, we remark the need for further studies that examine whether failure to upkeep the practice of emotion recognition and emotional expression would revert these effects, once more exacerbating the studied negative symptoms.

According to a recent study [39], emotion management may be associated with a change in amygdala–dorsolateral prefrontal cortex connectivity. As Guimond et al. has observed, this connectivity may provide a biological link to emotion management that can be enhanced [39]. From a biological perspective, this study’s EMP-S could affect this connectivity. Additionally, emotion management is associated with negative symptoms in patients with schizophrenia spectrum disorders [13], and emotion regulation and management positively impact autism spectrum disorder patients [40]; therefore, our EMP-S could also be modified to apply to these disorders.

### Limitations

First, the study included a very small sample with a one-group, pretest–posttest, quasi-experimental design as a pilot study. This is a significant limitation of this study; because there was no control group and participants were recruited from only one unit in one hospital, our results have limited generalizability. Although we applied GEE to control for potential confounders, we still strongly suggest the need for a future randomized controlled trial to investigate program effectiveness.

Second, we did not examine the longitudinal changes after the program completion; future studies are warranted to collect data four–six times during different periods, placing some of these after program completion, to allow for longitudinal examinations of program effects.

Third, a recent study [41] examined the effects of a virtual reality-based EMP in patients with schizophrenia; the world has been experiencing an unprecedented public health crisis owing to the COVID-19 pandemic, denoting the necessity to develop EMPs that employ various contactless forms of media. This would allow for the administration of such programs without major hinderances related to the social isolation policies imposed across several countries. Given this reality, and our lack of analyses on the topic during the current study, we remark the need for future research that taps into contactless EMPs.

Fourth, as shown here, the proposed EMP-S is anticipated to be effective in improving emotion recognition, emotional expression, and negative symptoms in an inpatient setting for patients with schizophrenia; therefore, subsequent studies should investigate the long-term effects of the program in various clinical settings.

## 5. Conclusions

Our results show that, even though the proposed EMP-S lasted half the time that prior existing interventions lasted for completion, it still showed improvements in emotion recognition and negative symptoms in patients with schizophrenia. Hence, the program is anticipated to be applicable for managing emotions and negative symptoms in patients with schizophrenia in modern hospital environments.

## Figures and Tables

**Table 1:** Description of the short emotional management program (EMP-S) developed in this study.

Session	Composition	Program Content	Time (min)
1	Emotional awareness and perception	1. Introducing the program and its participants	10
		2. Understanding the meaning of emotional words; understanding your emotions	30
		3. Sharing comments about the session and providing instructions about the next session	10
2		1. Connecting facial expressions and emotional words	10
		2. Recognizing emotions based on facial expressions and better understanding emotions in a particular situation	30
		3. Sharing comments about the session and providing instructions about the next session	10
3	Emotional expression	1. Expressing your basic emotions (i.e., fear, anger, hatred, sadness, joy, surprise)	10
		2. Role-playing through engaging in emotional expression during a conversation with another person	30
		3. Sharing comments about the session and providing instructions about the next session	10
4		1. Expressing emotions using instructions	10
		2. Role-playing through engaging in emotional expression in a group	30
		3. Sharing comments about the session and providing instructions about the next session	10
5	Emotional usage	1. Understanding others’ emotions	10
		2. Sharing others’ emotions using mood masks	30
		3. Sharing comments about the session and providing instructions about the next session	10
6		1. Understanding complex emotions	10
		2. Training the ability to convert emotions and finding happiness (1) Developing emotional inference skills (2) Giving hopeful and positive feedback to others	30
		3. Sharing comments about the session and providing instructions about the next session	10
7	Emotional regulation	1. Asking myself: Who am I?	10
		2. Developing resources and strategies needed to regulate emotions; designing a new future based on past experiences	30
		3. Sharing comments about the session and providing instructions about the next session	10
8		1. Recognizing and expressing depressed moods	10
		2. Recognizing and coping with negative emotions, such as anger and anxiety	30
		3. Wrapping up the program	20

**Table 2:** Participants’ general and disease-related characteristics (*n* = 17).

Characteristics	Categories	*n*	(%)	M(SD)
Sex	Men	6	(35.3)	
	Women	11	(64.7)	
Age (year)	20–29	2	(11.8)	45.82(10.11)
	30–39	3	(17.6)	
	40–49	2	(11.8)	
	50–59	10	(58.5)	
Marital status	Single	13	(76.5)	
	Married	4	(23.5)	
Education level	<College	8	(47.1)	
	≥College	9	(53.9)	
GAF scores	41–50	11	(64.7)	
	51–60	6	(35.3)	
Duration of disease (years)	<6 years	7	(41.2)	
	6–10 years	2	(11.8)	
	>10 years	8	(47.0)	
Duration of untreated psychosis	<1 month	17	(100)	
Antipsychotics dose ^†^	<600 mg	7	(41.2)	
	≥600 mg	10	(58.8)	
Benzodiazepine	no	6	(35.3)	
	yes	11	(64.7)	
Antidepressant	no	15	(88.2)	
	yes	2	(11.8)	
Mood stabilizer	no	9	(53.0)	
	yes	8	(47.0)	
Number of psychiatric hospital admissions	≤2	6	(35.3)	
	3–4	6	(35.3)	
	≥5	5	(29.4)	
Duration of employment	None	11	(64.7)	
	<2 years	3	(17.6)	
	≥2 years	3	(17.6)	

M, mean; SD, standard deviation; GAF, Global Assessment of Functioning Scale; ^†^ Chlorpromazine equivalents.

**Table 3:** Results for the comparisons between dependent variables and participants’ general and disease-related characteristics (*n* = 17).

Characteristics	Categories	*n*	Emotion Recognition M(SD)	*t*/F(*p*)	Emotional Expression M(SD)	*t*/F(*p*)	Negative Symptoms M(SD)	*t*/F(*p*)
Sex	Men	6	73.80(10.01)	0.30(0.768)	47.80(6.50)	1.92(0.783)	90.60(15.42)	0.90(0.384)
	Women	11	72.30(8.64)		54.80(6.75)		82.90(15.70)	
Age (year)	<46	6	77.67(7.28)	2.00(0.064)	48.00(4.78)	1.39(0.184)	92.67(10.07)	1.85(0.085)
	≥46	11	69.91(7.80)		53.27(8.50)		78.82(16.65)	
Marital status	Single	13	71.82(9.44)	0.71(0.493)	50.36(5.39)	2.06(0.060)	87.73(13.92)	0.93(0.368)
	Married	4	75.50(7.05)		58.25(9.47)		79.25(20.16)	
Education	<College	8	68.33(9.65)	1.72(0.110)	55.00(9.12)	1.10(0.287)	82.67(20.86)	0.56(0.587)
	≥College	9	75.78(7.21)		50.78(5.72)		87.33(11.79)	
GAF scores	41–50	11	72.00(9.98)	0.49(0.636)	49.70(4.92)	2.41(0.031) *	93.20(9.46)	3.85(0.002) **
	51–60	6	74.40(6.43)		58.00(8.57)		70.00(13.84)	
Duration of disease (years)	<6 years	7	70.83(9.33)	1.73(0.219)	51.17(4.26)	2.89(0.095)	89.83(11.27)	0.98(0.405)
	6–10 years	2	83.00(9.90)		44.00(5.66)		93.00(4.24)	
	>10 years	8	71.57(7.14)		56.00(7.94)		79.57(19.33)	
Duration of untreated psychosis	<1 month	17	72.80(8.79)		52.47(7.28)		85.47(15.51)	
Antipsychotics dose ^†^	<600 mg	7	75.51(8.32)	1.22(0.243)	52.14(6.54)	0.16(0.879)	85.29(10.86)	0.04(0.968)
	≥600 mg	10	70.25(8.89)		52.75(8.31)		85.63(19.49)	
Benzodiazepine	no	6	68.80(8.87)	1.27(0.225)	55.80(10.16)	1.28(0.222)	84.80(22.26)	0.11(0.911)
	yes	11	74.80(8.47)		50.80(5.22)		85.80(12.38)	
Antidepressant	no	15	73.77(9.02)	1.10(0.292)	52.46(7.54)	0.01(0.995)	85.46(14.51)	0.00(0.998)
	yes	2	66.50(3.54)		52.50(7.78)		85.50(28.99)	
Mood stabilizer	no	9	74.63(10.58)	0.85(0.410)	49.75(5.80)	1.64(0.126)	85.50(10.11)	0.01(0.993)
	yes	8	70.71(6.32)		55.57(7.96)		85.43(21.02)	
Number of psychiatric hospital admissions	<3	6	70.60(10.62)	0.67(0.513)	54.80(9.88)	0.87(0.400)	77.20(18.30)	1.53(0.150)
	≥3	11	73.90(8.12)		51.30(5.87)		89.60(12.97)	
Duration of employment	None	11	72.00(8.60)	0.58(0.575)	54.33(7.67)	1.64(0.235)	86.11(16.20)	0.76(0.490)
	<2 years	3	77.67(13.05)		46.00(5.56)		92.33(12.66)	
	≥2 years	3	70.33(5.13)		53.33(5.03)		76.67(16.86)	

M, mean; SD, standard deviation; GAF, Global Assessment of Functioning Scale scores; ^†^ Chlorpromazine equivalents. * *p* < 0.05, ** *p* < 0.01.

**Table 4:** Effects of the short emotional management program (EMP-S) on participants’ emotion perception, emotional expression, and negative symptoms (*n* = 17).

	95% Wald CI						
	*B*	SE	Lower	Upper	Wald χ^2^	*p*	*ES*
**Emotion recognition**							
Time					14.03	0.001 **	
Pre−test	0	0					
Two weeks into the program	3.07	2.09	−1.02	7.20	2.16	0.142	0.35
Post−test	7.00	2.60	1.91	12.09	7.25	0.007 **	0.73
a. Emotional attention							
Time					13.85	0.001 **	
Pre−test	0	0					
Two weeks into the program	1.53	1.14	−0.71	3.77	1.80	0.180	0.30
Post−test	3.27	1.18	0.96	5.57	7.72	0.005 **	0.63
b. Emotional clarity							
Time					6.52	0.038 *	
Pre−test	0	0					
Two weeks into the program	1.67	1.37	0.33	4.34	1.49	0.222	0.31
Post−test	3.73	1.74	−1.01	7.14	4.62	0.032 *	0.65
**Emotional expression**							
Time					4.15	0.126	
Pre−test	0	0					
Two weeks into the program	1.79	1.48	−1.12	4.69	1.46	0.046 *	0.13
Post−test	3.66	1.83	0.07	7.24	4.00	0.227	0.36
**Negative symptoms**							
Time					115.27	<0.001 ***	
Pre−test	0	0					
Two weeks into the program	−24.20	3.11	−30.30	−18.10	60.38	<0.001 ***	1.18
Post−test	−44.87	4.31	−53.32	−36.42	108.31	<0.001 ***	2.10
a. Affective blunting							
Time					103.83	<0.001 ***	
Pre−test	0	0					
Two weeks into the program	−6.00	0.97	−7.90	−4.10	38.21	<0.001 ***	0.95
Post−test	−12.80	1.27	−15.29	−10.31	101.16	<0.001 ***	1.84
b. Alogia							
Time					133.50	<0.001 ***	
Pre−test	0	0					
Two weeks into the program	−4.93	0.67	−6.25	−3.62	54.25	<0.001 ***	1.00
Post−test	−8.80	0.86	−10.49	−7.11	104.71	<0.001 ***	1.62
c. Apathy							
Time					66.78	<0.001 ***	
Pre−test	0	0					
Two weeks into the program	−4.27	0.91	−6.04	−2.49	22.15	<0.001 ***	1.26
Post−test	−7.60	0.95	−9.46	−5.74	63.83	<0.001 ***	2.33
d. Asociality							
Time					81.28	<0.001 ***	
Pre−test	0	0					
Two weeks into the program	−5.73	1.07	−7.83	−3.64	28.79	<0.001 ***	1.23
Post−test	−10.20	1.14	−12.43	−7.97	80.61	<0.001 ***	2.49
e. Attention							
Time					48.68	<0.001 ***	
Pre−test	0	0					
Two weeks into the program	−3.27	0.69	−4.62	−1.92	22.45	<0.001 ***	1.14
Post−test	−5.47	0.79	−7.02	−3.91	47.44	<0.001 ***	1.79

Note. Covariates: GAF scores; SE, standard error; CI, confidence interval; GAF, Global Assessment of Functioning Scale scores; *p* value: GEE model adjusted for covariates; ES: effect size (Cohen’s d). * *p* < 0.05, ** *p* < 0.01, *** *p* < 0.001.

## Data Availability

The data presented in this study are available on request from the corresponding author and with permission from the Institutional Review Board of Eulji University.

## References

[1] James S.L., Abate D., Abate K.H., Abay S.M., Abbafati C., Abbasi N., Abbastabar H., Abd-Allah F., Abdela J., Abdelalim A. (2018). Global, regional, and national incidence, prevalence, and years lived with disability for 354 diseases and injuries for 195 countries and territories, 1990–2017: A systematic analysis for the Global Burden of Disease Study 2017. Lancet.

[2] Huang C.L., Hsiao S. (2017). The functional significance of affect recognition, neurocognition, and clinical symptoms in schizophrenia. PLoS ONE.

[3] Bonfils K.A., Lysaker P.H., Minor K.S., Salyers M.P. (2017). Empathy in schizophrenia: A meta-analysis of the interpersonal reactivity index. Psychiatry Res..

[4] Painter J.M., Stellar J.E., Moran E.K., Kring A.M. (2019). A multicomponent approach toward understanding emotion regulation in schizophrenia. J. Clin. Psychol..

[5] Hargreaves A., Mothersill O., Anderson M., Lawless S., Corvin A., Donohoe G. (2016). Detecting facial emotion recognition deficits in schizophrenia using dynamic stimuli of varying intensities. Neurosci. Lett..

[6] Abramowitz A.C., Ginger E.J., Gollan J.K., Smith M.J. (2014). Empathy, depressive symptoms, and social functioning among individuals with schizophrenia. Psychiatry Res..

[7] Lora A., Kohn R., Levav I., McBain R., Morris J., Saxena S. (2012). Service availability and utilization and treatment gap for schizophrenic disorders: A survey in 50 low- and middle-income countries. Bull. World Health Organ..

[8] Kucharska-Pietura K., Mortimer A. (2013). Can antipsychotics improve social cognition in patients with schizophrenia?. CNS Drugs.

[9] Hutchison S.L., MacDonald-Wilson K.L., Karpov I., Maise A.M., Wasilchak D., Schuster J.M. (2017). Value of psychiatric rehabilitation in a behavioral health Medicaid managed care system. Psychiatr. Rehabil. J..

[10] Blanchard J.J., Cohen A.S. (2006). The structure of negative symptoms within schizophrenia: Implications for assessment. Schizophr. Bull..

[11] Kirkpatrick B. (2014). Developing concepts in negative symptoms: Primary vs secondary and apathy vs expression. J. Clin. Psychiatry.

[12] Beck A.T., Rector N.A., Stolar N., Grant P. (2008). Schizophrenia: Cognitive Theory, Research, and Therapy.

[13] Yolland C.O.B., Carruthers S.P., Toh W.L., Neill E., Sumner P.J., Thomas E.H.X., Tan E.J., Gurvich C., Phillipou A., Van Rheenen T.E. (2020). The relationship between negative symptoms and both emotion management and non-social cognition in schizophrenia spectrum disorders. J. Int. Neuropsychol. Soc..

[14] Castro V.L., Cheng Y., Halberstadt A.G., Grühn D. (2016). EUReKA! A conceptual model of emotion understanding. Emot. Rev..

[15] Kring A.M., Elis O. (2013). Emotion deficits in people with schizophrenia. Annu. Rev. Clin. Psychol..

[16] Cho H., Gonzalez R., Lavaysse L.M., Pence S., Fulford D., Gard D.E. (2017). Do people with schizophrenia experience more negative emotion and less positive emotion in their daily lives? A meta-analysis of experience sampling studies. Schizophr. Res..

[17] Park S.A. (2018). Study on the theory of mind deficits and delusions in schizophrenic patients. Issues Ment. Health Nurs..

[18] Kolavarambath R., Sudhir P.M., Prathyusha P.V., Thirthalli J. (2020). Emotion recognition, emotion awareness, metacognition, and social functioning in persons with schizophrenia. Indian J. Psychol. Med..

[19] Kee K.S., Horan W.P., Salovey P., Kern R.S., Sergi M.J., Fiske A.P., Lee J., Subotnik K.L., Nuechterlein K., Sugar C.A. (2009). Emotional intelligence in schizophrenia. Schizophr. Res..

[20] Marsh P.J., Polito V., Singh S., Coltheart M., Langdon R., Harris A.W. (2016). A quasi-randomized feasibility pilot study of specific treatments to improve emotion recognition and mental-state reasoning impairments in schizophrenia. BMC Psychiatry.

[21] Won M.R., Lee K.J., Lee J.H., Choi Y.J. (2012). Effects of an emotion management nursing program for patients with schizophrenia. Arch. Psychiatr. Nurs..

[22] Javed A., Charles A. (2018). The importance of social cognition in improving functional outcomes in schizophrenia. Front. Psychiatry.

[23] Cho M., Jang S.J. (2019). Effect of an emotion management programme for patients with schizophrenia: A quasi-experimental design. Int. J. Ment. Health Nurs..

[24] Mechanic D. (2007). Mental health services then and now. Health Aff..

[25] Salinsky E., Loftis C. (2007). Shrinking inpatient psychiatric capacity: Cause for celebration or concern? Issue brief. Georg. Wash. Univ. Natl. Health Policy Forum.

[26] Peritogiannis V., Gioti P., Gogou A., Samakouri M. (2020). Decrease of hospitalizations and length of hospital stay in patients with schizophrenia spectrum disorders treated in a community mental health service in rural Greece. Int. J. Soc. Psychiatry.

[27] Fenger M., Mortensen E.L., Poulsen S., Lau M. (2011). No-shows, drop-outs and completers in psychotherapeutic treatment: Demographic and clinical predictors in a large sample of non-psychotic patients. Nord. J. Psychiatry.

[28] Cha S.G. (2009). Development and Effectiveness of the Emotion Management Program for Chronic Schizophrenic Patients. Ph.D. Thesis.

[29] Salovey P., Mayer J.D., Goldman S.L., Turvey C., Palfai T.P., Pennebaker J.W. (1995). Emotional attention, clarity, and repair: Exploring emotional intelligence using the trait meta-mood scale. Emotion, Disclosure, & Health.

[30] Gross J.J., John O.P. (1995). Facets of emotional expressivity: Three self-report factors and their correlates. Pers. Individ. Dif..

[31] Kupper N., Duijndam S., Karreman A. (2020). Emotion expressivity in Dutch: Validation of the Dutch translation of the Berkeley expressivity questionnaire. Psychol. Assess..

[32] Andreasen N.C. (1989). Scale for the assessment of negative symptoms (SANS). Br. J. Psychiatry Suppl..

[33] Preda A., Nguyen D.D., Bustillo J.R., Belger A., O’Leary D.S., McEwen S., Ling S., Faziola L., Mathalon D.H., Ford J.M. (2018). A positive take on schizophrenia negative symptom scales: Converting scores between the SANS, NSA and SDS. Schizophr. Res..

[34] Usher K., Woods C., Parmenter G., Hutchinson M., Mannix J., Power T., Chaboyer W., Latimer S., Mills J., Siegloff L. (2017). Self-reported confidence in patient safety knowledge among Australian undergraduate nursing students: A multi-site cross-sectional survey study. Int. J. Nurs. Stud..

[35] Tsotsi S., Kosmidis M.H., Bozikas V.P. (2017). Improved facial affect recognition in schizophrenia following an emotion intervention, but not training attention-to-facial-features or treatment-as-usual. Psychiatry Res..

[36] Park E.J., Park J.G. (2016). The effect of role-playing focused emotion management training for chronic schizophrenia patients on their emotional expression and interpersonal relationship functioning. Cogn. Behav. Ther. Korea.

[37] Zou Y.M., Ni K., Yang Z.Y., Li Y., Cai X.L., Xie D.J., Zhang R.T., Zhou F.C., Li W.X., Lui S.S.Y. (2018). Profiling of experiential pleasure, emotional regulation and emotion expression in patients with schizophrenia. Schizophr. Res..

[38] Granholm E., Harvey P.D. (2018). Social Skills Training for Negative Symptoms of Schizophrenia. Schizophr. Bull..

[39] Guimond S., Ling G., Drodge J., Matheson H., Wojtalik J.A., Lopez B., Collin G., Brady R., Mesholam-Gately R.I., Thermenos H. (2020). Functional connectivity associated with improvement in emotion management after cognitive enhancement therapy in early-course schizophrenia. Psychol. Med..

[40] Factor R.S., Swain D.M., Antezana L., Muskett A., Gatto A.J., Radtke S.R., Scarpa A. (2019). Teaching emotion regulation to children with autism spectrum disorder: Outcomes of the stress and anger management program (STAMP). Bull. Menn. Clin..

[41] Souto T., Silva H., Leite A., Baptista A., Queirós C., Marques A. (2020). Facial emotion recognition: Virtual reality program for facial emotion recognition—A trial program targeted at individuals with schizophrenia. Rehabil. Couns. Bull..

